# Melanin: a unifying theory of disease as exemplified by Parkinson’s, Alzheimer’s, and Lewy body dementia

**DOI:** 10.3389/fimmu.2023.1228530

**Published:** 2023-09-29

**Authors:** Stacie Z. Berg, Jonathan Berg

**Affiliations:** Department of Translational Biology, William Edwards LLC, Baltimore, MD, United States

**Keywords:** Parkinson’s disease, Lewy body dementia, Alzheimer’s disease, melanin, glyphosate, inflammation, amyloid plaque, gut microbiome

## Abstract

Melanin, a ubiquitous dark pigment, plays important roles in the immune system, including scavenging reactive oxygen species formed in response to ultraviolet radiation absorption, absorbing metals, thermal regulation, drug uptake, innate immune system functions, redox, and energy transduction. Many tissue types, including brain, heart, arteries, ovaries, and others, contain melanin. Almost all cells contain precursors to melanin. A growing number of diseases in which there is a loss of melanin and/or neuromelanin are increasingly thought to have infectious etiologies, for example, Alzheimer’s disease (AD), Parkinson’s disease (PD), Lewy Body Dementia (LBD), and vitiligo. AD, PD, LBD, and vitiligo have been linked with herpesvirus, which enters melanosomes and causes apoptosis, and with gut dysbiosis and inflammation. Herpesvirus is also linked with gut dysbiosis and inflammation. We theorize that under normal healthy states, melanin retains some of the energy it absorbs from electromagnetic radiation, which is then used to fuel cells, and energy from ATP is used to compliment that energy supply. We further theorize that loss of melanin reduces the energy supply of cells, which in the case of AD, PD, and LBD results in an inability to sustain immune system defenses and remove the plaques associated with the disease, which appear to be part of the immune system’s attempt to eradicate the pathogens seen in these neurodegenerative diseases. In addition, in an attempt to explain why removing these plaques does not result in improvements in cognition and mood and why cognitions and moods in these individuals have ebbs and flows, we postulate that it is not the plaques that cause the cognitive symptoms but, rather, inflammation in the brain resulting from the immune system's response to pathogens. Our theory that energy retained in melanin fuels cells in an inverse relationship with ATP is supported by studies showing alterations in ATP production in relationship to melanin levels in melanomas, vitiligo, and healthy cells. Therefore, alteration of melanin levels may be at the core of many diseases. We propose regulating melanin levels may offer new avenues for treatment development.

## 1 Introduction

Dementia is characterized by cognitive difficulties, memory loss, poor judgment, confusion, agitation, and apathy (1). Alois Alzheimer, and a short time later, Fritz Heinrich Lewy, linked the characteristic plaques in the brain with dementias (2, 3), which later became known as Alzheimer’s disease (AD) and Parkinson’s disease (PD), respectively. These plaques generally continue to be thought of as causative, despite that no drug or other treatment targeting these plaques has been found to adequately treat, stop the progression of, or cure these diseases. We are at a standstill for a theory as to what might be happening in the individuals with neurodegenerative and other diseases. If AD, PD, and Lewy Body Dementia (LBD), are not caused by plaques in the brain, what are these neurodegenerative diseases caused by?

Overlooked in neurodegenerative and other diseases are the roles of melanin. Melanin is a pigment mostly known for absorbing the genetically-damaging ultraviolet (UV) radiation from the sun and releasing it as heat and is part of the immune system. Typically dark in color (eumelanin and neuromelanin), neuromelanin increases as individuals age (4), but significant loss is seen in individuals with AD, PD, and LBD (5–7). Melanin also has antioxidant properties; it reduces reactive oxygen species (ROS) (8). Hence, we postulate that elevated oxidative stress seen in these neurodegenerative diseases is related to a loss of melanin/neuromelanin. Mitochondrial dysfunction is also seen in these diseases (9, 10), and there is an increasing amount of research linking elevated oxidative stress seen in neurodegenerative diseases with mitochondrial dysfunction (11). However, there is still an exceedingly important missing factor, and that is melanin has electron storage and transport capabilities and appears to provide energy to cells in an inverse relationship with that supplied by mitochondria.

Microbes also have been generally overlooked as part of the etiological puzzle. However, various brain disorders that affect cognitions, including AD, PD, LBD, multiple sclerosis (MS), and amyotrophic lateral sclerosis (ALS), have been associated with infection, and the plaques in AD, PD, and LBD brains have been found to be encapsulating these pathogens. Hence, the plaques may be part of a normal immune system response to the presence of pathogens (12) but are not clearing in the brains of individuals with these diseases. This leads to the question of why?

We theorize that the brain’s immune system’s inability to clear the plaques is due to the loss of melanin. Specifically, we focus on the energy-storing role of melanin, as we theorize it and its electron storage and transport capabilities to be at the foundation of disease. We use here AD, PD, LBD, vitiligo, and melanoma, all of which are characterized by a loss (AD, PD, LBD, and vitiligo) or increase (melanoma) in the levels of melanin, as models for our theory that the presence, loss, or increase of melanin can be used to explain key underlying mechanisms of health and disease. More recently, a link to the gut microbiome and consequential inflammation also has been recognized as contributory factors to these neurodegenerative diseases (11), which is supportive of our theory, as seen later in this hypothesis/theory paper. Importantly, as noted earlier, these neurodegenerative diseases are increasingly being associated with pathogens. One common link among these diseases is herpesvirus (13–18), which we use here for our model. Interestingly, and not coincidentally in our view, vitiligo, which is characterized by a lack of melanin, is also associated with herpesvirus (19), gut dysbiosis (20), and increased risk of developing dementia (21).

In sum, AD, PD, LBD, and vitiligo are all linked with a loss of melanin and gut dysbiosis (20–22). Herpesvirus is also linked with gut dysbiosis, dementia (23), and vitiligo (loss of melanin) (18). Gut dysbiosis leads to an immune response and inflammation (24). This should not be surprising, because the vast majority, 70 percent to 80 percent of immune cells, are located in the gut, and intestinal microbiota and the local mucosal immune system interact. In addition to these local mucosal immune responses, the gut microbiome affects systemic immunity (25).

The purpose of this paper is to provide evidence from the scientific literature of a link between an alteration in melanin levels and disease and to attempt to explain how the alterations could occur and their effects on disease as well as melanin’s possible role as a storage molecule for energy used by cells to fuel the immune system and for other biological functions. Consideration should be given to the idea that melanin may play a vital role in supplying cellular energy and a significant protective role in diseases anywhere in the body where it is found, providing a unified theory of disease, which extends to cardiovascular disease, cancer, vitiligo, epilepsy/seizures, neuroimmune diseases, neurodegenerative diseases, other diseases of the nervous system, other diseases of the gastrointestinal tract, mast cell diseases, autoimmune diseases, connective tissue disorders, diseases related to adipose tissues, diseases related to epithelial cells, including arteries, veins, capillaries, heart, brain, lungs, and/or muscles, diabetes, diseases involving ears, eyes, nose, and/or skin, and other diseases, disorders, and conditions relating to any form of melanin and/or its precursors, and/or mitochondria (**Table 1**). Further, in our view, it is the inflammation associated with neurodegenerative diseases (32) that causes the changing cognitive status and mood symptoms, and we think that it is a result of fluctuations in inflammation in response to the peaks and wanes in immune system response to infection. Further, we suggest it is a reduction in barometric pressure, which naturally occurs as the sun sets and lasts until about 10 p.m.(33), that allows the brain inflammation to increase and causes sundowning in individuals with dementia where sundowning is seen.

**Table 1:** Known and proposed functions of melanin and diseases associated with melanin.

Functions of melanin	Functions of melanin proposed by the authors	Diseases associated with alteration in melanin levels	Diseases proposed by the authors to be associated with alteration in melanin levels	References
UV radiation absorption, photoprotection, pigmentation, metals absorption, drug uptake and retention, reactive oxygen species (ROS) scavenger/antioxidant, thermoregulation, innate immune system roles, transduction of energy, involved in redox	Possible primary supplier of energy to cells to fuel cellular processes. Supplies energy in inverse relationship with mitochondria.	Vitiligo, albinism, Parkinson disease, Alzheimer’s disease, Lewy Body Dementia, melanoma, others	Neurodegenerative, neuroinflammatory, and neuroimmune diseases, including AD, PD, LBD, MS, ALS, vascular dementia, traumatic brain injury/chronic inflammatory encephalopathy, other brain disorders, including epilepsy/seizures, other diseases of the nervous system, autoimmune diseases, diseases of the gastrointestinal tract, diabetes, cancer, cardiovascular diseases, including heart disease, stroke, deep vein thrombosis, chronic venous insufficiency, diseases related to epithelial cells, including those involving arteries, veins, capillaries, the brain, heart, lungs, and/or muscles, mast cell diseases, connective tissue disorders, diseases related to adipose tissues, acute and long COVID-19 and other post-infectious autoimmune encephalopathies, neuropsychiatric disease, including depression, post traumatic stress disorder, mood disorders, bipolar disorder, schizophrenia, and other neuropsychiatric illnesses, autism, attention deficit hyperactivity disorder, Down syndrome, and other developmental disorders, insomnia, drug addiction, diseases involving ears, eyes, nose, and/or skin, and other diseases, disorders, and conditions related to or involving any form of melanin and/or its precursors and/or mitochondria	(4–8, 19, 26–31)

## 2 Infection and the building of amyloid plaques

Research over more than three decades has found the beta amyloid (ß-amyloid/Aβ) plaques in the brains of individuals with AD contained infectious microbes, most notably, herpes simplex virus type 1 (HSV1). In addition to HSV1, other pathogens had been linked with AD: *Chlamydia pneumoniae*, several types of spirochaete, and fungal infection (34). A recent study of 500,000 medical records expanded on this work, linking influenza, herpesviruses, and Epstein-Barr, among other viral infections, to AD, PD, MS, ALS, generalized dementia, and, interestingly, vascular dementia (35), which further supports our theory, as vasculature, specifically endothelial cells, also contain melanin.

As researchers in Europe were isolating microbes from the beta amyloid plaques found in the brains of individuals with AD, in the U.S., Australian researcher Robert Moir had the idea that the ß-amyloid plaques in the AD brain were remnants of the immune system killing pathogens. In 2016, he and his colleagues published their first *in vivo* model of ß-amyloid’s role as an antimicrobial (36). They had discovered the antimicrobial activity in Aβ to be 100 times more potent than penicillin for some pathogens. Most important for AD is that Moir found that Aβ captures and kills pathogens by the binding of Aβ clumps to the microbial cell surface, which triggers the formation of amyloid fibrils. The fibrils interfere with the microbes attaching to host cells, thereby preventing infection. The growing fibrils entangle the pathogen and pull it into a structure, agglutinate, where the pathogen becomes temporarily neutralized. The fibrils poke holes, forming pores in the surface of the pathogen, and then the contents of the pathogen cell leak out. When a fibril forms from Aβ, the protein’s affinity for copper ions increases 1 billion times. Aβ draws in copper ions from the local environment and uses that metal to catalyze chemical reactions that produce high levels of hypochlorite ion (bleach). That sterilizes the aggregate and causes the amyloid fibrils to fuse in a process called cross-linking. In addition, it stops Aβ from producing more bleach in order to prevent damage to nearby brain tissue, and it makes the fibrils resistant to enzymatic degradation. This is important because the pathogens secrete various enzymes to degrade the Aβ fibrils, which allows them to escape. However, the brain’s local clearinghouse cells are not able to easily remove Aβ and agglutinate, which can remain for decades (12, 37).

Since Moir’s discovery, evidence of accumulation of Aβ inside neurons has been found. This process happens prior to neurofibrillary tangles formation and extracellular Aβ (reviewed in Bayer and Wirths) (38). We suspect the same amyloid fibril encapsulation and bleaching process may take place inside neurons that have been attacked by pathogens. This bleaching could explain the reduction in neuromelaninin in AD and in other neurodegenerative diseases in which amyloid plaques that produce bleach are present, as they are in PD and LBD (39). Alternatively, we theorize the pathogens are using the melanin for their own purposes, possibly to bolster their own immune systems or their attacks on the host and/or using the energy to multiply, and/or for building and fortifying shelters while depleting stores for use by the host. More specifically, regarding the latter, we postulate the extracellular matrix is an immune system defense, and the intracellular matrix is made by the pathogens, and the pathogens are using the melanin to fuel manufacturing the matrix and, potentially, using the neurons as a structure to hide in.

Could it be that in individuals with neurodegeneration, the brain no longer has the energy to clear what might be a normal response to pathogen entry? It is possible that because glial cells, which remove plaque, pathogens, and other debris (40), contain melanin (41), and melanin is reduced or depleted, there is not enough melanin to fuel them, hence, the plaques and encapsulated pathogens remain. One of the things we found profoundly interesting is that people with these dementias, especially PD and LBD, have periods of time when their memories are accessible and cognitions improved. In fact, fluctuations in cognitive ability and alertness are part of the symptomology (42). Administration of certain drugs also results in improvement, sometimes immediate and profound, as in the case of nilotinib (43–45). The ebbs and flows of neurological symptoms suggests to us that the pathways are obstructed by inflammation that peaks and wanes early on in an immune system response; however, affected neurons remain intact, but atrophied (46), and, aside from the missing melanin, otherwise healthy in AD (6), which gives hope that patients’ neurological functioning can be restored, if we identify the true underlying cause of these neurodegenerative diseases and treat it effectively.

## 3 Melanin: the unifying theory of disease

**Figure 1:**
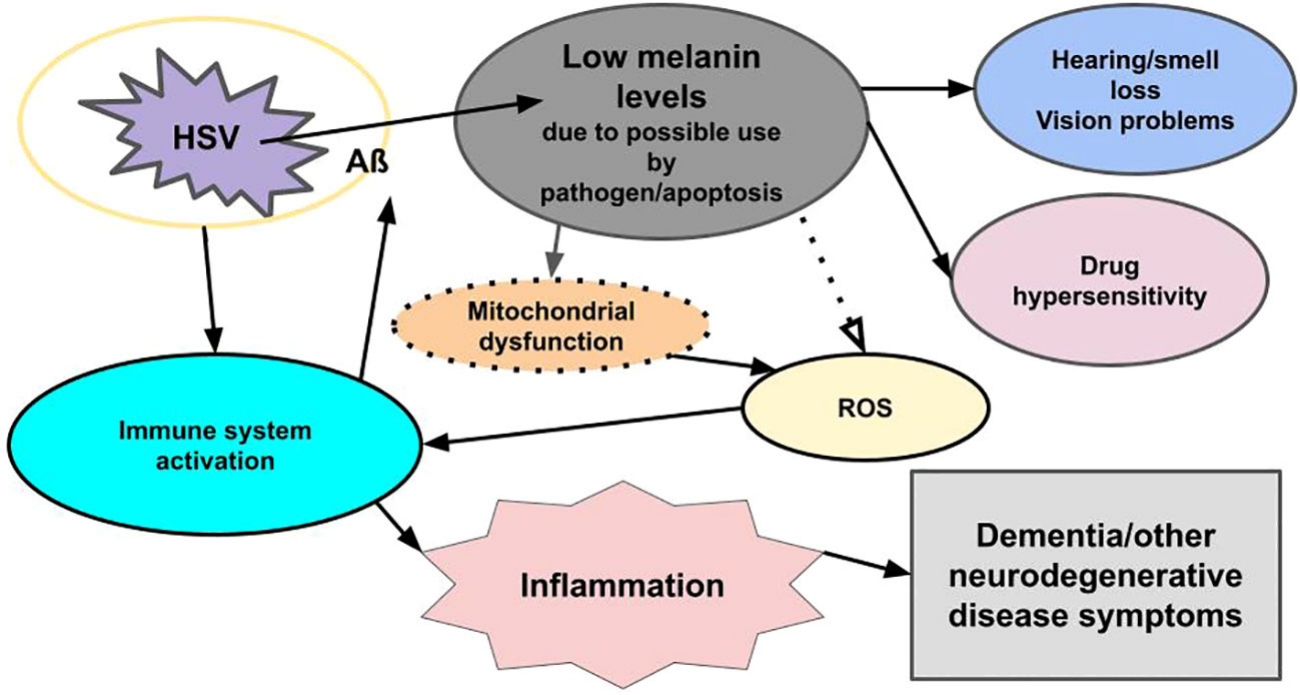
Viral-related neural inflammation and neurodegeneration. Infection with a microbe (for example, HSV) triggers an immune response, which leads to the formation of an encapsulating plaque (Aß) and inflammation, while reducing melanin levels through apoptosis and/or usurpation by pathogens. The loss of melanin triggers mitochondrial dysfunction, which leads to increased ROS, which triggers an immune response, which triggers inflammation. Low melanin levels/apoptosis also cause hearing/smell loss, vision problems, and drug hypersensitivity. Loss of melanin also may be seen in the skin (not shown). The pathogen also triggers the release of mast cells in an immune response (not shown). Mast cells degranulate and release histamine and other immune system inflammatory chemicals. The immune response to both the pathogen and reduced melanin levels lead to inflammation, and the pathogen triggers apoptosis, which reduces melanin and dopamine (not shown), all of which lead to dementia and other neurodegenerative disease symptoms.

We theorize the key to neurodegenerative and other diseases lies in melanin, specifically, the energy that we postulate melanin supplies, and that the reduction in neuromelanin seen in neurodegenerative diseases results in the inability of the brain to clear the immune system of plaques, which are encapsulating pathogens (**Figure 1**). If this is true, then it would not be surprising to see the immune system take an “all hands on deck” approach in the areas of the brain undergoing depletion of the near-black melanin and produce higher concentrations of pheomelanin, a reddish pigment, because pheomelanin absorbs more of the shorter, higher frequency, higher energy, wavelengths of the light spectrum compared with the dark eumelanin/neuromelanin, and less pheomelanin relative to eumelanin is required for absorbing ultraviolet (UV) photons (47), which are considerably more energetic than visible light. In 2023, Cai et al. (48) found just that in the substantia nigra of individuals with PD, the area of the brain affected by this disease. Further, nilotinib, the drug with such remarkable videoed patient responses in 2015 (no longer available on the internet) that caused such excitement in the field amongst both researchers and patients, was not only shown to help clear the brain of plaques in individuals with LBD, PD, and AD, but it also crosses the blood-brain barrier (43–45) and induces melanogenesis (49).

We now must ask the question of if melanin holds energy that the brain, and body, particularly the immune system, require, and parts of the immune system itself require, how do we know that? Further, how can we explain in the context of energy the exhausting tremors that characterize PD when there is a reduction of melanin in the brains of individuals with this disease? First, we know that in healthy individuals, neuromelanin accumulates with age (4), which suggests to us a need for more energy intake and/or improved immune system functioning as we age. However, in individuals with neurodegenerative diseases, there is a loss of dark melanin in specific areas of the brain, depending on the disease, and that absence correlates with disease symptomatology (50, 51). This led us to the conundrum of explaining the exhausting resting tremors in PD. If melanin contains energy, then energy would need to be conserved in a disease state, not spent excessively, which it would appear resting tremors would do. However, Amano et al. (52) provide a theoretical framework that demonstrates that resting tremor and other motor behaviors seen in PD are actually metabolically energy efficient. They posit that the role of dopamine, which is a precursor to the formation of neuromelanin, and energy metabolism in the brain is linked and supported this assertion with research finding that “dopamine lesions result in reduced glucose uptake,” showing a preservation of energy, and dopamine is related to glucose metabolism. They conclude, “the loss of dopamine neurons in PD is likely to contribute to dysfunctional glucose metabolism.” They also note the growing amount of literature arguing mitochondrial dysfunction is common in PD and is causing metabolic dysfunction. We have discovered that there is an inverse relationship between melanin levels and mitochondrial ATP production. In fact, it appears to us that melanin may hold the primary supply of energy while mitochondria produce supplemental energy, in an opposing, but complimentary, interdependent relationship.

## 4 ATP production compliments energy from melanin

We began our search for evidence of the possible role of melanin in supplementing cellular fuel by reviewing the literature on mitochondria in vitiligo cells. Vitiligo cells are characterized by a lack of pigmentation (53). We also looked at malignant melanoma cells, which are characterized by a proliferation of melanocytes, and, therefore, typically, increased melanin.

Dell’Anna et al. (26) compared cultured epidermal vitiligo melanocytes to healthy controls. They found that compared with normal cells, epidermal vitiligo melanocytes were low in ATP production. The cells compensated for low energy through an increased uptake of glucose, an increased production of certain enzymes used to metabolize glucose, and increased volume and mass of the mitochondria. This hints at the possibility that these cells actually may have increased ATP production to compensate for the loss of energy that we postulate melanin supplies to healthy (non-vitiligo) cells and that the ATP may not have been captured in the measurements, because of its rapid expenditure in the absence of melanin. It is possible that the increased size of the mitochondria could allow for more capacity to produce ATP, and the increased glucose uptake and increased enzymes could be used to fuel the production of ATP in the absence of energy typically supplied by melanin. In addition, in vitiligo, epidermal melanocytes are not expending any of their energy producing melanin, which should actually conserve energy, increasing the availability of ATP.

If our theory is correct, then it would follow that melanoma cells would have an abundance of energy. Hall et al. (27) determined that melanoma cells had impaired energetic metabolism as a result of dysfunctional oxidative phosphorylation. Whereas normal cells get most of their ATP from oxidative phosphorylation, the malignant melanoma cells obtained more of their ATP from glycolysis. Interestingly, interference with glycolysis in these malignant melanoma cells did not cause all of the malignant melanoma cells to die, whereas interference with oxidative phosphorylation in normal melanocytes resulted in the death of all cells (27), suggesting the possibility that the surviving malignant melanoma cells, in addition to glycolysis, were obtaining energy from another source, melanin, as only cells with an abundance of melanin survived (**Table 2**). In addition, malignant melanoma cells need increased energy due to their genetic programming to proliferate. In other cancers, oxidative phosphorylation is not impaired; on the contrary, mitochondria biogenesis and mitochondrial quality control are known to be upregulated in most other cancers (54). Therefore, it is possible that malignant melanoma cells obtain energy from the abundance of melanin.

**Table 2:** Inverse relationship between melanin and ATP production theory.

	Vitiligo Cells	Melanoma Cells	References
**Known characteristics**	Increase in uptake of glucose, increase in production of certain enzymes used to metabolize glucose, increase in volume and mass of the mitochondria	Decrease in oxidative phosphorylation and obtain more ATP from glycolysis than from oxidative phosphorylation	(26, 27)
**Theory**	We postulate that vitiligo cells have increased ATP production as a result of the lack of melanin.	We postulate that melanoma cells have decreased ATP production as a result of increased energy from melanin. When the main source of ATP production was inhibited in normal and melanoma cells, all normal cells died but not all melanoma cells died.	(27)

**Figure 2:**
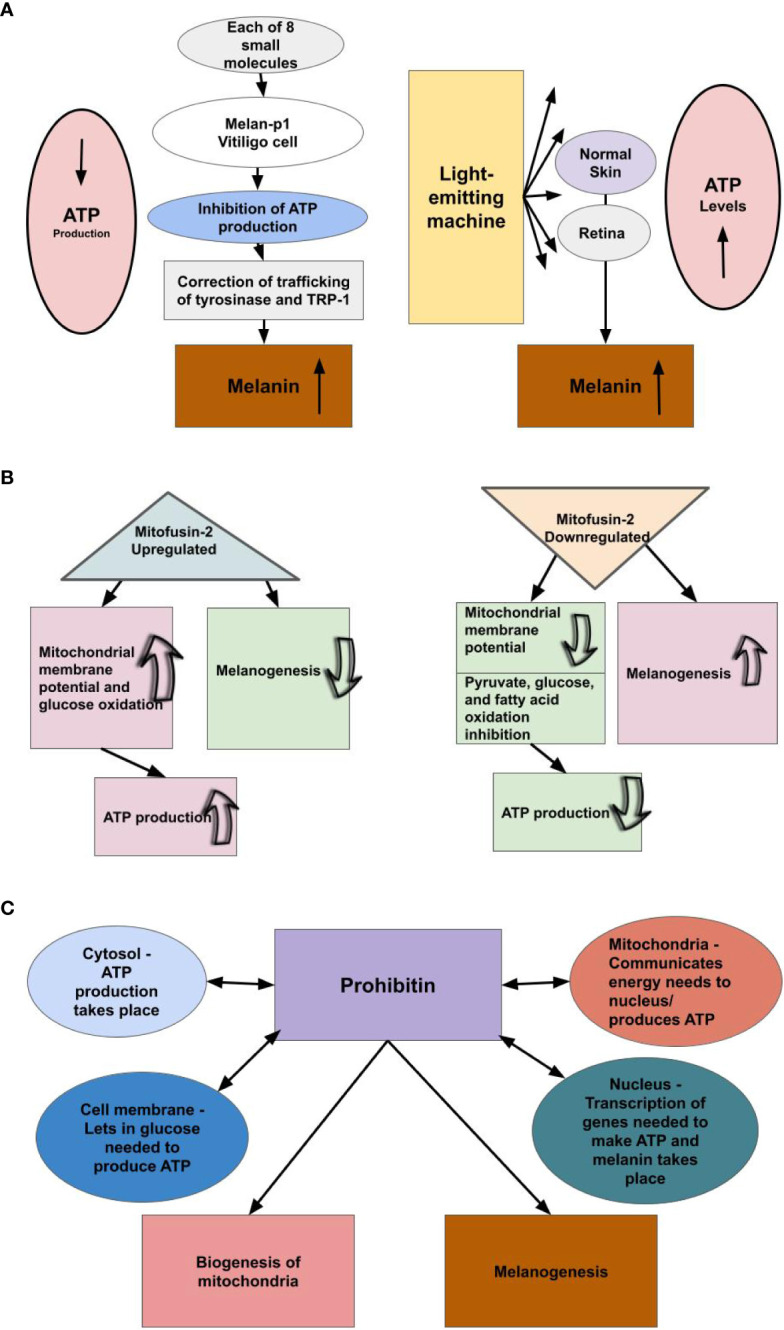
We theorize there is an inverse relationship between melanin levels and ATP production. **(A)** Small molecule inhibitors of mtATPase, which inhibit mitochondrial ATP production, increase melanin production by a factor of three-fold or greater in melan-p1 cells. Therefore, melanin levels rise when the ATP pathway is blocked. Light-emitting equipment increases levels of cellular ATP, we theorize, because not as much ATP is being used as a result of increased availability of melanin/neuromelanin. Light is not a precursor to ATP production; however, light is a precursor to melanogenesis. Therefore, we conjecture that when pigmented cells were exposed to the emitted light, they produced melanin/neuromelanin. Hence, it appears ATP levels rise when cells that produce melanin/neuromelanin are exposed to light. This suggests that ATP production and melanin production are inversely related. **(B)** Upregulating the production of Mitofusin-2 increases ATP production, while decreasing the rate of melanogenesis, and downregulation of the production of Mitofusin-2 decreases ATP production, while increasing the rate of melanogenesis. **(C)** Prohibitin is a protein found in the mitochondria, nucleus, cell membrane, and cytosol and shuttles amongst them. We theorize prohibitin is communicating energy needs among these cellular components to produce ATP and balancing that with its known roles of mitochondrial biogenesis and melanogenesis, the latter of which we theorize is providing the primary energy to cells.

Further evidence that melanin may be involved in supplying energy to cells can be found in the link between the inhibition of ATP production by mitochondria and the increased production of melanin. Williams et al. (28) identified six small molecules that increased melanin production threefold or greater in melan-p1 cells. All six of those small molecules were found to inhibit mitochondrial F1F0-ATP synthase (mtATPase). After identifying those six small molecules, two additional small molecules known to inhibit mtATPase were tested and, remarkably, also found to increase melanin production – and it was by a similar amount. The researchers proposed that these eight small molecules increased melanin production by “inhibiting mtATPase-mediated transport of H^+^ ions,” which altered pH, and the alteration of the pH led to an increase in melanin production by correcting the trafficking of tyrosinase, the enzyme that catalyzes the rate-limiting step of melanogenesis, and tyrosine-related protein-1 (TRP-1) to the proper compartments (**Figure 2A**). This shows one way of how inhibiting mitochondrial ATP production can induce an increase in melanin production – by causing a change in pH, which thereby corrects trafficking of certain proteins critical to the process of melanogenesis.

We propose the reason for the inverse relationship between melanin levels and ATP production is that melanin supplies energy to cells. If melanin provides energy to cells, then increasing melanin increases the amount of energy that cells are obtaining from melanin. Therefore, it would follow that the body evolved in such a way that molecules that cause a great increase in melanin over an individual’s baseline, thus providing extra energy to the cell, would also limit mitochondrial ATP production, as this ATP would be wasted. Due to its low stability in water, ATP cannot be stored in cells, except for very short periods of time (55). Many studies have found that the use of light-emitting equipment leads to increased cellular ATP levels. It is not yet understood why this occurs (56). However, it is important to note that these studies measured cellular ATP levels, not cellular ATP production. We theorize that melanin is converting some of the emitted light into energy, and cells are able to utilize this energy instead of ATP. Thus, we theorize that there was not an increase in ATP production, as was assumed, and the reactions did not take place in the mitochondria. Instead, we suggest the measured increase in cellular ATP levels was due to a reduction in ATP usage due to an increase in energy produced by melanin, as a result of absorption of the light, which the cells were then able to utilize, conserving ATP (**Figure 2A**). This also points to our conclusion that energy from melanin is the first choice of energy for a large number of cellular pathways and provides further explanation for the inverse relationship between melanin levels and levels of mitochondrial ATP production. As noted previously, ATP has low stability in water, and, therefore, can only be stored in cells for short periods of time (55). Thus, if energy from melanin is the first choice for many pathways, then if mitochondrial ATP production does not decrease much, there will be an overabundance of ATP in the cells, as seen in these studies. Much of this ATP may end up degrading and, therefore, being wasted. This explains the evolutionary benefit to decreasing mitochondrial ATP production when there is an increase of melanin.

We found further evidence showing the inverse relationship between mitochondria ATP production and melanogenesis. Pich et al. (57) found that downregulation of Mitofusin-2 (Mfn2) results in the inhibition of pyruvate, glucose, and fatty acid oxidation and the reduction of mitochondrial membrane potential, while upregulation of Mfn2 results in an increase of glucose oxidation and mitochondrial membrane potential. This is important to note, as Tanwar et al. (58) found that downregulating Mfn2 increases the rate of melanogenesis, while upregulating Mfn2 decreases the rate of melanogenesis. Furthermore, they also found that heavily pigmented human primary melanocytes had a lessened expression of Mfn2 compared with lightly pigmented human primary melanocytes (59). This suggests an inverse relationship between melanin production and mitochondrial ATP production, as downregulation of Mfn2 decreases oxidative phosphorylation and increases melanogenesis, while upregulation of Mfn2 increases oxidative phosphorylation and decreases melanogenesis, thereby supporting our belief that melanin supplies energy to the cell (**Figure 2B**). Furthermore, Bach et al. (59) found that Mfn2 is downregulated in obese individuals. This further supports the inverse relationship between mitochondrial ATP production and melanin, as adipose tissue contains melanin, and the adipose tissue of obese individuals contains more melanin than the adipose tissue of lean individuals (60), and it is known that there is mitochondrial dysfunction in the adipose tissue of obese individuals (61). Further, when neuromelanin in dopaminergic neurons accumulated in PD animal models exceeded a threshold, it interfered with mitochondrial function along with other cell processes. Specifically, mitochondria consumed less oxygen, which is predictable under our model (4).

Next we looked at prohibitin (PHB), a protein found in the mitochondria, nucleus, cell membrane, and cytosol. It is known to be shuttled from one part of the cell to another (62). Its functions include biogenesis of mitochondria, and other important functions within the mitochondria (63), and melanogenesis by affecting the rate of transcription of the gene that encodes tyrosinase, the rate-limiting enzyme of melanogenesis (64). It is interesting to consider that cells produce ATP in their mitochondria and cytosol, the mitochondria communicate with the nucleus about energy needs (65), and the nucleus is where transcription takes place, and proteins in the cell membrane let materials in and out of the cell, which include the glucose needed to produce ATP. Thus, the four parts of the cell where PHB is found and where it is shuttled between are all involved in energy production (**Figure 2C**). It is also interesting to note that the eight small molecules that were determined by Williams et al. (28) to increase pigmentation by a factor of threefold or greater also affected tyrosinase, in these cases rerouting it. We think it is possible that PHB signaling may be being used to trigger the production of melanin, when necessary.

While we began our search with the idea of melanin supplementing ATP production, in the end, we realized it is the other way around.

### 4.1 The relationship between melanin production and ATP production in neurodegenerative disease

While the details are not clear, cholesterol appears to be related to AD, as do triglycerides. For example, Moser et al. (66) found an association between fluctuations in total cholesterol and triglycerides, which are stored energy, and the incidence of AD and other dementias. This provides further support for a relationship between melanogenesis and ATP production: 1. Cholesterol regulates melanogenesis in a time- and dose-dependent manner (67); and 2. The breakdown of triglycerides (lipids) form ATP in a process called lipolysis (68).

Alcohol is another form of energy (69). Researchers recently found in mouse models that excessive alcohol intake may accelerate the progression of AD (70). In humans, chronic alcohol intake is associated with impairment of ATP synthesis (71) and hyperpigmentation (72).

In neurodegenerative disease, ATP levels are low (73). However, this does not mean ATP production is low. We postulate ATP is being used more rapidly in neurodegenerative diseases as a result of low levels of melanin production and its associated energy. This led us to investigating if there is an inverse relationship between ATP production and melanin in terms of neurodegenerative diseases, which we suspected there would be, and we did, in fact, find evidence showing an inverse relationship between melanin and ATP in studies on nicotine’s affect on melanin production. Delijewski et al. (74) treated melanocytes with various concentrations of nicotine for 24 hours. Nicotine at the lowest concentrations had no affect on melanin production. However, in cells treated at the middle concentrations, melanin concentrations increased 26 percent and 15 percent, reducing as concentrations rose. At the highest concentration, melanin content was reduced by about 16 percent. Next, they looked at cellular tyrosinase activity in melanocytes also treated with various concentrations of nicotine for 24 hours and found it correlated with the effects on melanogenesis. The lowest nicotine concentrations had no effect. At the middle concentrations, tyrosinase activity rose by about 26 percent and 16 percent, respectively, reducing as concentrations rose. At the highest concentrations, tyrosynase activity was reduced by 14 percent. Although there are no studies explicitly looking at ATP production, melanin production, and nicotine, we would expect to see an increase in ATP when melanin production is low and a decrease in ATP when melanin production is high. What we found was telling: exposure to cigarette smoke resulted in a dose-dependent inhibition of mitochondrial complex I and complex II and a reduction in mitochondrial respiration and ATP production (75). Another study showed exposure to cigarette smoke decreases mitochondrial respiration, and, consequently, decreases ATP levels and increases oxidant production in a dose-dependent manner (reviewed in Fetterman) (76). When levels of ATP production decrease, electron carriers are not oxidized as frequently, thereby resulting in the formation of superoxide, a major species of ROS (77). Thus, we predict a graph comparing melanin levels to ROS levels would be a U-shaped curve, with ROS increasing as melanin decreases from baseline, due to there being less melanin to scavenge the ROS (8, 77), and with ROS also increasing as melanin increases from baseline, as production of melanin creates ROS (78). Increases in melanin result in a decrease in ATP production, which also increases ROS, as detailed previously (77).

Because nicotine at certain concentrations increases melanin, it would follow, then, according to our theory, that an increase in nicotine at those concentrations would prevent or delay the onset of PD and AD, and potentially, treat it. In fact, more than 70 studies demonstrate that the use of tobacco reduces the risk of developing PD (reviewed in Nicholatos) (79). The risk of developing AD decreased as the number of cigarettes smoked daily increased prior to disease onset, which the researchers theorize is due to nicotine (80). Nicotine is thought to be a major mediator of neuroprotection (81), and that finding was further elucidated by Nicholatos et al. (79). The reduction of PD risk by tobacco is dependent on the duration and intensity of use (82). Nicotine administration can improve cognitive impairment in AD and in PD can improve dyskinesia and memory (reviewed in Alhowail) (83) and gait and reduce falls (84). Nicotine appears to accumulate in cells containing melanin, which may increase melanin synthesis (85). We think that this may have important implications for a better understanding of the immune system and of melanin and energy, as there are multiple entryways to the brain.

### 4.2 Electrical charge and electromagnetic synthesis

From the electrical stimulus that makes the heart beat to electrical signals transmitting information intercellularly through electrical waves, currents are continuously flowing throughout the human body. Overlooked is the role melanin plays in the flow of electricity. Melanin molecules typically bind in a chain-like formation. This structure allows for electron flow (86). Melanin also stores (45) and, thereby, acts as a bank for, electrons, and it conducts electricity (85, 87).

In addition to its ability to conduct, store, and release energy in the form of electricity (86, 88), melanin has the ability to display bistable switching. Melanin is an electronic-ionic hybrid conductor, not an amorphous organic semiconductor, as was previously thought (87), and can turn on and off under different voltages (86). This on-off switch may be a mechanism used in its relationship with the energy produced by mitochondria.

Now that we know melanin holds an energy supply, vital to the immune system, as discussed later, we need to know where it can be found and what it does there. In addition to the eyes, hair, skin, ovaries, heart, and arteries, including those leading into the heart, melanin is present in other organs and tissues, including the liver, inner ear, lung, connective tissues, spleen, kidney, testes, peritoneum, muscles, choroid, pineal gland, and, of course, the brain (dopaminergic and noradrenergic neurons, meninges covering the brain, and glial cells) (41, 89). In fact, melanin may exist throughout the body. We found in our search of GeneCards database that the genes encoding tyrosinase, TRP-1, and dopachrome tautomerase (TRP-2), enzymes that are part of the melanin biosynthesis pathway, are expressed in nearly all types of organ tissues. Therefore, all cells might have the ability to produce melanin. Another way to view the flow of electricity throughout the body is in sickness. If the amount of electricity becomes erratic or surges, there is a disease state, such as arrhythmias or epilepsy. And when there is a dramatic reduction in electron flow, and, consequently, a significant loss of energy, we postulate that is when and how neurodegenerative diseases form, as neuromelanin supplies the brain with energy, and with the absence of energy, neurodegenerative diseases can develop when pathogens are present.

In fact, this has been demonstrated in another organ, the heart, and in the epileptic brain. Levin et al. (90) found that murine hearts with cardiac melanocyte-like cells lacking TRP-2 were less pigmented and more susceptible to abnormal electrical activity compared with the hearts of control mice, which we would expect if any melanin precursor was reduced or missing. Furthermore, they found that murine cardiac melanocyte-like cells were coupled electrically to adjacent myocytes (90), which suggests melanin provides an energy source to heart muscle. Earlier we noted that vascular dementia is also linked to infection (35). Vascular dementia has similar characteristics to AD, PD, and LBD: the arteries contain plaque, and the epithelial cell lining of the arteries, veins, and capillaries are pigmented with melanin (91, 92), which we theorize provides energy. We suggest researchers determine if the epithelial cells of individuals with vascular dementia have reduced melanin levels in the vasculature of their brains and/or heart or in the area of a deep vein thrombosis or other cardiac event that may have occurred, increased lipids, and mitochondrial dysfunction.

**Figure 3:**
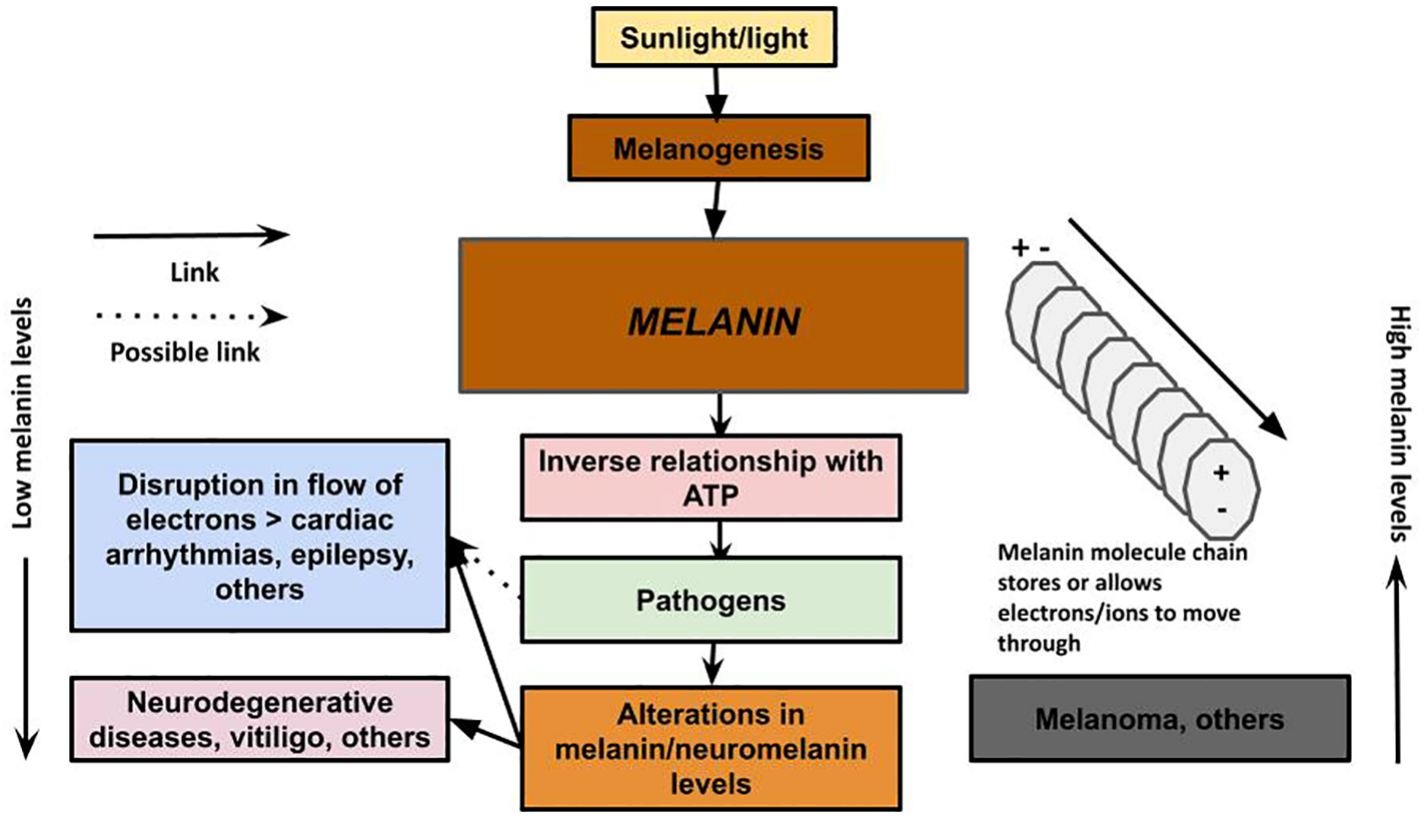
Melanin has the ability to conduct, store, and release energy. Melanin: The unifying theory of disease describes a new understanding of the role of melanin as a primary energy supplier to cells, with an inverse relationship with ATP production, and provides a new theory on the pathology of neurodegenerative and other diseases. We propose that in neurodegenerative disease, the loss of melanin/neuromelanin reduces cellular energy in the brain, which interferes with the immune system’s ability to clear the plaques encapsulating microbes that may have migrated from the gut via the vagus nerve, nose via the olfactory nerve, or via some other pathway. The loss of melanin also can lead to disruption of electrical impulses that can result in cardiac arrhythmias and seizures. Excessive melanogenesis can fuel diseases, such as melanoma.

Phenylalanine hydroxylase catalyzes the conversion of phenylalanine to tyrosine. Approximately 75 percent of phenylalanine from diet and protein catabolism gets converted to tyrosine (93). This is important, because tyrosine, and, hence, phenylalanine, is a precursor to melanin (94), and the symptoms of phenylalanine hydroxylase deficiency appear, to us, to be related to low melanin levels similar to PD: Parkinson-like symptoms, musty odor (95), and intellectual disability (cognitive problems). Also seen is reduced pigmentation of skin and hair, a clear sign of low levels of melanin, and women with a history of recurrent miscarriage and/or offspring with malformations, which could imply not having enough energy to maintain a pregnancy and fully form a fetus. Epilepsy, with its electrical surges, is also related to phenylalanine hydroxylase deficiency (96), and, hence, an abnormally low level of melanin. Our model would predict that these low levels of melanin would force a high production of ATP, which would cause surges in electrical activity (seizures) as a result of malfunctioning mitochondria. In fact, there is mitochondrial dysfunction in epilepsy as well as oxidative stress (97), which we would predict would happen when there is not enough melanin to scavenge the ROS, and there is a dramatic increase in ATP concentrations in epilepsy seizures (98), supporting our hypothesis (**Figure 3**).

## 5 Properties of melanin

Peripheral melanin is produced in the epidermal layer of the skin. Melanin scavenges ROS formed in response to UV radiation (29), absorbs metals, thermoregulates, is involved in drug uptake, is part of the innate immune system, transduces energy (reviewed in Koike et al.) (30), and is involved in redox. It is expected to have many other functions and properties yet to be identified (29). Pathogenic microorganisms, including bacteria and fungi, use melanin to protect against defense mechanisms from infected hosts (99).

Melanin is a polymer. The production of melanin is triggered by UV radiation. Melanin is produced in organelles called melanosomes within melanocytes (100). Melanosomes are then transported into keratinocytes where they cluster around the nucleus to protect its DNA from UV radiation (30). Melanocytes interact with several systems. Hence, melanogenesis is controlled by the immune, endocrine, inflammatory, and central nervous systems. Melanocyte activity is also regulated by extrinsic factors, including UV radiation, as noted previously, and certain drugs (100).

One of the most fundamental aspects of our hypothesis lies in the fact that light is made up of photons, packets of energy (101) that have no size. Therefore, the pathway to organ tissue, even those protected by bones, is wide open from the perspective of a photon. Hence, we theorize, that just as the melanin in skin absorbs UV radiation from the sun, so, too, does melanin everywhere throughout the body, including neuromelanin in the brain, and this may be of utmost importance when it comes to the future treatment of neurodegenerative diseases.

### 5.1 Melanogenesis pathway

**Figure 4:**
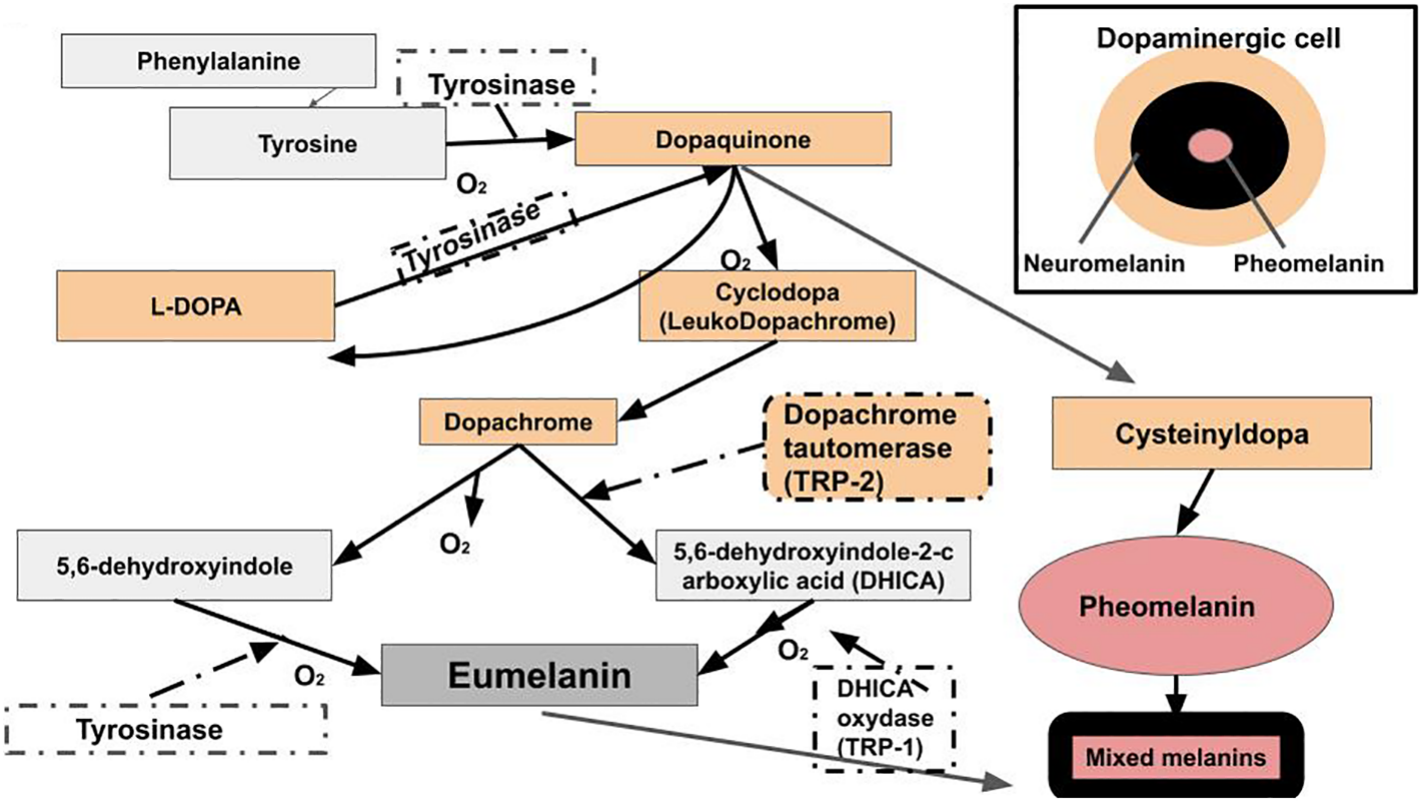
Raper-Mason melanogenesis pathway. Eumelanin, pheomelanin, and neuromelanin involve L-DOPA. L-DOPA is a precursor to dopamine. Dopamine plays a major role in PD, LBD, and AD.

The exact makeup of melanin is unknown. Its chemical structures are diverse and complex (29, 87). Melanogenesis takes place in the melanosome and begins when L-phenylalanine hydroxylase catalyzes the reaction that converts phenylalanine to the amino acid tyrosine (94). Tyrosine is converted into dopaquinone by the enzyme tyrosinase. Dopaquinone can be converted into L-3,4-dihydroxyphenylalanine (L-DOPA). L-DOPA can be converted back into dopaquinone by a second reaction with tyrosinase. Dopaquinone is a precursor to both eumelanin and pheomelanin. Dopaquinone undergoes a conversion into leukodopachrome. This produces another L-DOPA molecule, which can be converted back into dopaquinone. Leukodopachrome is converted to dopachrome. Dopachrome can be decarboxylated to generate 5,6-dihydroxyindole, which reacts with tyrosinase to produce eumelanin. Dopachrome interacts with the enzyme TRP-2. This converts the dopachrome into DHICA (5,6-dihydroxyindole-2-carboxylic acid). The 5,6-dihydroxyindole and DHICA molecules are polymerized together. The polymerization of these monomers is catalyzed by TRP-1 into eumelanin. Melanogenesis for pheomelanin starts with tyrosine, which gets converted to L-DOPA. L-DOPA gets converted to 5-cysteinyldopa, and 5-cysteinyldopa gets converted to pheomelanin (29, 87, 102–104) (**Figure 4**). The structure and synthesis pathway of neuromelanin remains mostly unknown (105). Neuromelanin, which contains pheomelanin at its core (97), is produced in neuronal cells and accumulated in both catecholaminergic neurons, specifically, dopaminergic and noradrenergic neurons, and glial cells (105). Hence, it is derived from dopamine or norepinephrine. Therefore, the production of eumelanin, neuromelanin, and pheomelanin involves L-DOPA. L-DOPA is a precursor to dopamine. Dopamine plays a major role in PD (106), LBD (107), and AD (108).

## 6 Melanin and virulence

Specific pathogens are known to attack specific cell types for specific reasons. For example, *Helicobacter pylori* colonizes gastric epithelial cells to control the immune system so that they can proliferate (109). Therefore, it is interesting to ponder why certain pathogens attack cells containing melanin. We have conceptualized two theories: 1. Melanin is a vital part of the human immune system, and ridding the cell of melanin weakens the host. 2. Pathogens may be pirating host melanin to enhance their own defenses. Melanization in pathogens has been linked with their virulence. Pigment in infectious microbes often increases their virulence by interfering with immune clearance mechanisms of the host or by initiating proinflammatory or cytotoxic properties. For example, melanin is found in *Cryptococcus neoformans* and *Aspergillus fumigatus*, which can cause severe opportunistic infection in patients with compromised immune systems (110). Due to its negative charge, melanin interferes with the action and efficacy of endogenous antimicrobial peptides and antifungals used to treat infection from *C. neoformans* (111). It would, therefore, benefit a microbe to alter host DNA to produce more melanin. It is interesting to note that melanoma increases the risk of PD fourfold, and PD increases the risk of melanoma fourfold (112). Forty-one different bacteria in melanoma samples were identified in one study (113). We suggest both diseases are caused by the same pathogen in affected individuals.

Whereas melanization in pathogens has been linked with their virulence (110), inhibition of melanization reduces pathogen virulence (114). The same was found to be true for the microbe: the less melanin, the more susceptible it is to being killed by ROS or UV radiation (115). For example, melanin-lacking mutants of *C. neoformans* were shown to be less virulent (116, 117). Furthermore, melanin-deficient mutants were found to be less able to resist phagocytic killing *in vitro* (118). Melanin may control inflammatory responses via T cells, as well. A heavily melanized strain of *C. neoformans* inhibited T-cell immune response compared with a lesser pigmented strain (119).

Certain viruses can trigger inhibition of melanogenesis in infected tissue of human hosts by killing or disabling melanocytes. Herpesviruses (120), cytomegalovirus, a herpesvirus (121), human immunodeficiency virus (HIV), chronic hepatitis C virus (122), and hepatitis B virus (123) have been associated with vitiligo. Koike et al. (30) suggest that toll-like receptor-3 (TLR-3) activation (which promotes melanosome transfer and extracellular release of melanosomes) caused by viral infection could contribute to vitiligo pathogenesis by both melanocyte apoptosis and the suppression of melanin synthesis as a result of melanocyte dysfunction. TLRs are receptors that recognize molecular patterns of microbes. They are classified as Interleukin-1 receptors (124). Varicella-zoster virus, a herpesvirus, also can lead to depigmentation in melanocytes (125). A study by Harson et al. (126) showed that the primary site of viral replication in the skin in the early stages of infection by Varicella-zoster virus in vitiligo could be melanocytes wherein the virus highjacks the intracellular mechanism of pigment formation for viral replication. It appears to us that in addition to escaping the immune system properties of melanin, it may be that pathogens use the melanin from their host to fortify their own supply. We posit that, similar to vitiligo, pathogens may trigger apoptosis in cells containing neuromelanin. We posit here, too, that in addition to escaping the immune system properties of melanin, they may be destroying host melanin to reduce immune system defenses or using host melanin to fortify their own immune systems.

In the case of PD and LBD, drug hypersensitivity, tinnitus, and loss of olfactory sense all arise in tissues that contain melanin. It is interesting to note that olfactory sense degrades prior to symptoms of dementia in LBD and in AD. It is thought in AD to be due to entry of an infectious microbe through the nose, migration up the olfactory nerve, and movement into the brain, as described by Itzhaki (34), and it is not farfetched to think that this pathway may be the same for PD and LBD. We suggest replication of the pathogen takes place in the melanosomes of the nose, and, possibly, the ears, whereupon apoptosis occurs. Loss of neuromelanin is found in AD, PD, and LBD (5–7). Further, LBD is also thought to be correlated with a reduction in skin melanin (127). Interestingly, vitiligo increases the risk of developing any form of dementia (21). As previously noted, herpesvirus is linked with vitiligo (120). Herpesvirus induces apoptosis in melanocytes (128). We theorize that the reduction in olfactory sense and the loss of hearing result from apoptosis of these melanized cells, and through an unelucidated mechanism, dampens those sensory outlets.

Pathogens may not only find easy transport to the olfactory nerve and to the ear but also to the eyes, as it is not uncommon for individuals to rub their eyes with unclean hands. Therefore, it is possible that pathogens enter through the eyes. Likewise, they may travel internally to that area. Regardless, there is often significant visual involvement in dementias. AD, PD, and LBD have all been associated with visual disturbances. Visuoperceptual disturbances in PD include problems with face and facial emotion recognition, color discrimination, contrast sensitivity, and overlapping object identification (129). All of these dementias have been associated with posterior cortical atrophy, a neurodegenerative syndrome that presents with decline in visuospatial and visuoperceptual skills as well as literacy and praxic skills (130). Also seen is thinning of the inner retinal layer, retinal dopaminergic deficiency, and cortical thinning in the posterior cortex (129, 131). Further, melanin in the uveal tract is thought to improve visual acuity by absorbing light (41). If there is a loss of melanin in any of these melanized areas, it would help to explain visual disturbances and reduction in literacy and apraxic skills. In fact, dopaminergic cell loss has been reported in the retina in PD (132). Dopaminergic cells contain neuromelanin (41, 105). Neuromelanin has been identified in the cortex (133). It is also possible, from our perspective, that some of the visual and literacy and praxis skill changes could be due, in part, to connective tissue problems in the uvea and elsewhere caused by mast cells, as seen in strabismus in Ehlers-Danlos syndrome (134). Strabismus, which is related to convergence insufficiency, responds to dopamine replacement therapy in PD (135). The loss of melanin would imply a loss of dopamine.

## 7 Glyphosate, the gut, and the effects of melanin loss

Another way to show melanin’s effect in neurodegenerative and other neuroimmune diseases is to look at the effects of the herbicide glyphosate. Glyphosate inhibits melanogenesis in humans and in pathogens (136). In the context of what we present here about melanin and its role in disease, it is not surprising that glyphosate has been linked with PD and AD (137).

In addition to inhibiting melanogenesis in humans, glyphosate kills microbes on plants that certain human gut flora need by blocking the shikamate pathway of these microbes. Puigbò et al. (138) found that the shikimate metabolic pathway is present in 54 percent of common gut bacteria in humans and, therefore, they are intrinsically sensitive to glyphosate. Barnett and Gibson (139) note that opportunistic pathogens compared to commensal bacteria are more resistant to glyphosate. Glyphosate-induced dysbiosis is linked with inflammation (140, 141). Zhang et al. suggest soluble melanin or melanized bacteria may prevent gut inflammation and dysbiosis (142).

The shikimate pathway is responsible for producing tyrosine, phenylalanine, and tryptophan, the three aromatic amino acids used in protein synthesis. These amino acids are upstream of other important biological molecules, including pigment compounds. Metabolites derived from these aromatic amino acid pathways are involved in nervous signal transmission and quell ROS in the brain. Humans and other animals do not have the shikimate pathway. However, they rely on a diet of both plants and microbes to prevent gut dysbiosis and its consequences (143).

It is important to note that phenylalanine and tyrosine are precursors to melanin synthesis (93), and aromatic amino acids obtained from the diet are upstream of the biosynthesis of neurotransmitters. Phenylalanine and tyrosine are also precursors to the biosynthesis of L-DOPA, dopamine, norepinephrine, and epinephrine. Tryptophan is a precursor for the synthesis of another neurotransmitter, serotonin, as well as melatonin (a neurohormone), niacin (a vitamin), the neuroprotectant kynurenine, and NAD^+^ and NADP^+^ (enzyme cofactors) (143), most of which are affected in neurodegenerative diseases.

In insects, as well, glyphosate has been shown to inhibit the synthesis of melanin. Smith et al. (144) concluded that glyphosate exposure could increase susceptibility of insects to pathogens as a result of melanin inhibition and immune system impairment.

If a loss of melanin is a keystone of symptoms in neurodegenerative disease, then increasing melanin may provide a novel treatment. Melanogenesis would also logically improve symptoms or eradicate the disease. As noted previously, nilotinib, an anti-cancer drug, induces melanogenesis (145). Administration of nilotinib in patients with AD, PD, or LBD showed a reduction in cognitive and motor symptoms (43–45). The same is true of nicotine, which increases melanin at certain doses, and cognitive symptoms (74, 75, 84).

## 8 Melanin, histamine, and the immune system

We postulate that with the loss of melanin seen in neurodegenerative diseases, it may be that the body tries to compensate by attempting to synthesize melanin. Research shows that histamine concentrations were found to be significantly increased in PD patients (146). Histamine activates tyrosinase, which triggers melanogenesis (147). Increased histamine levels also have been found in the basal ganglia of individuals with PD (148). Histamine also causes neuroinflammation (149). In AD, neuronal histaminergic system changes predict cognitive decline, and histamine 3 receptor (H3R) antagonists alleviate cognitive symptoms (150). Raised levels of H3R in cortical and basal ganglia structures were found in individuals with dementia with Lewy bodies and AD. An increase in H3R in the globus pallidus was associated with delusions and visual hallucinations (151). We theorize that inflammatory aspects of high histamine levels is an underlying cause of dementia. Importantly, and we think this is key, Kawamoto et al. (152) found that melanin acts as a potent inhibitor of mast cell degranulation. Histamine is produced and stored in mast cells. Loss of neuromelanin would allow histamine levels to rise. Rocha et al. (153) found that histamine induces microglial activation and dopamanergic cell death. The effects of melanin treatment for allergic response was approximately equal to that of ketotifen fumarate and without evidence of cytotoxicity. The study provides evidence for use of homogenized melanin as a possible therapeutic agent for diseases involving mast cells (152), and it further supports our theory.

Interestingly, melanin ingested from foods impacts the immune system. ElObeid et al. (31) reviews the enhancement and modulation of the immune system by melanin through ingestion, including release of IgA and Interleukin-1, cytokine production, increased antibody production, and other immune system response activation as well as anti-inflammatory and antioxidant effects and other protective effects.

## 9 Adverse drug reactions and melanin

Another question to ask is what is the connection between neurodegenerative diseases and drug hypersensitivity, and is it connected to melanin? Individuals with dementia are at increased risk of adverse effects of drugs that act on the central nervous system (154), and we think the loss of melanin in neurodegenerative disease may be causing these drug hypersensitivities, as certain drugs have a high binding capacity for tissues containing melanin (155). In fact, data from Bridelli et al. (156) demonstrate melanin’s capacity to bind with drugs to be comparable to medicinal activated charcoal. Melanin and neuromelanin may be retaining drugs and metals to protect cells and then slowly release them, but build up and release may cause toxicity (31). The retention of drugs in pigmented tissues of the inner ear, eyes, and skin damage cells. Chloroquine, for instance, accumulates in dermal melanocytes and hair follicles, where it can cause tinnitus, hearing loss, and dizziness (156). In addition, some drugs mediate skin phototoxicity (157). High neuromelanin affinity appears to play a role in the adverse drug reactions in the central nervous system and the etiology of PD (158).

## 10 Evidence that light improves neurodegenerative disease

If our theory is correct, it would follow that delivering photons into the areas of the brain where neuromelanin is in short supply would effectively treat neurodegenerative and other diseases. Photobiomodulation is a technology that uses light therapeutically to heal the body. It is used transcranially to treat brain disorders. The mechanisms of photobiomodulation are not well understood. It is thought that chromophores in the mitochondria absorb the photons. These chromophores include cytochrome C oxidase, opsins, heat-gated ion channels, and cytochromes/flavoproteins. However, there is no strong evidence for any of these chromophores absorbing significant amounts of light, and there is evidence against cytochrome C oxidase playing a role in light absorption. Regardless, photobiomodulation has shown efficacy in treating various brain diseases, including neurodegenerative disease by clearing the plaques, reducing inflammation, and restoring mitochondrial functioning (reviewed in Hamlin) (159). We theorize that the light is being absorbed by melanin.

Interestingly, as will be further explored in the next section, there are different microglia phenotypes. Neuroinflammation results from activation of microglia in the M1 phenotype, which release pro-inflammatory cytokines with down regulated phagocytic functionality. These microglia scavenge for pathogens, plaques, and damaged neurons. Microglia can shift via photobiomodulation from the M1 phenotype to the M2 phenotype, which clears plaques and exerts anti-inflammatory and anti-oxidant effects. Photobiomodulation also has been shown to stimulate neurogenesis and promote synaptogenesis, which is vital for treating neurodegenerative disorders as well as traumatic brain injuries, stroke, and mood disorders (reviewed in Hamblin) (159).

## 11 Stem cells restore pigmented neurons and mitochondrial function

If our theory is correct and the problem underlying neurodegenerative disease is pathogenic entry into neuronal cells and subsequent immune response, including inflammation, but a lack of energy to clear the pathogens, new, healthy cells would treat the disease. In fact, research on stem cells has provided important evidence for that being the case. Transplantation of human endometrial-derived stem cells into an immunocompetent PD mouse model restored dopamine production (160). This suggests formation into dopaminergic cells, which contain melanin (41, 105). Mesenchymal stem cells from umbilical cords, bone marrow, and adipose tissue induced microglial activation and reduced Aβ deposition, tau hyperphosphorylation, and neuroinflammation in AD mouse models, resulting in improved cognitive functioning (161–164). Human menstrual blood-derived stem cell (MenSC) intracerebral transplantation into the brains of AD mouse models resulted in microglia activation with a phenotypic change that caused expression of anti-inflammatory cytokines rather than the pro-inflammatory cytokines that had been present. These newly-formed microglia removed Aβ plaques (165). The work by Moir et al. (12) illustrates how these plaques encapsulate and kill pathogens. We theorize that the new, healthy cells provided by the MenSCs removed the pathogens as a result of having a normal energy supply from melanin and suggest that the induction of the non-inflammatory microglia may have resulted from the eradication of the pathogens. Further, in AD, tau is typically hyperphosphorylated in neurons, which causes tau to detach from the microtubules. That detachment causes mitochondria not to be transported to the axons. The MenSCs were predicted to have caused tau to no longer be hyperphosphorylated (165), allowing transport of the mitochondria into the axons and restoring mitochondrial function.

## 12 Testing the theories

To investigate melanin’s role in neurodegenerative diseases and develop new treatments and treatment protocols, we envision, in order to provide more immediate solutions, repurposing drugs and technologies already approved for use in other indications. Research for efficacy and safety is necessary. One approach for consideration could be: 1. Test blood for indications of immune responses to pathogens. Note that not all infections may show up in these measurements. 2. Administer an anti-inflammatory that crosses the blood brain barrier but does not affect melanin/melanogenesis. 3. Remove extracellular and intracellular plaques and matrices with drugs already on the market for this purpose. 4. Kill the pathogens with appropriate intracellular antimicrobials that cross the blood-brain barrier and that are able to penetrate any matrix and fibril debris. One antimicrobial we think is worthy of investigation for future use, due to its potency and its ability to break up biofilms, is high molecular mass hyaluronic acid derived from naked mole-rats, which may have the added benefit of preventing, and possibly, removing plaque. Interestingly, naked mole-rats synthesize large amounts of high molecular mass hyaluronic acid of mass 6-12 MDa in brain and other tissue types, and naked mole-rats do not grow plaques in their brains. This high molecular mass hyaluronic acid is substantially heavier than the high molecular mass hyaluronic acid that humans synthesize (1-2 MDa) (166–168). 5. Improve neuromelanin levels, trialing drugs, including nicotine, especially the patch to avoid oral exposure, nilotinib, and salicylamide, which also increases melanin (169, 170), as well as homogenized melanin, or melanin derived from foods in high concentrations. Light technology, such as an intensified version of the one developed for the NICU by Cincinnati Children’s Hospital (171), reconstructed to disperse wavelengths in an appropriate light spectrum for triggering melanin/neuromelanin/pheomelanin production, or other photobiomodulation using external light boxes or other devices or methodologies that can be developed or altered to radiate appropriate light wavelengths to induce melanin/neuromelanin/pheomelanin production, should also be explored. In addition, the eight small molecules that increase melanin in melan-p1 cells (28) and other novel strategies to repair melanocytes and/or promote melanogenesis should be investigated. Finally, a test to determine normal and abnormal melanin levels should be developed not only for predicting and monitoring AD, PD, and LBD, but for all diseases and conditions in which melanin may play a role and/or for those diseases with mitochondrial dysfunction.

### 12.1 A cautionary statement

We caution against using a treatment that increases melanin without eradicating the pathogens first, because an increase in melanin could potentially fuel the pathogenic process.

## 13 Conclusion

We think there is sufficient evidence in the scientific literature to support our theories that 1. under normal healthy states, melanin retains some of the energy it absorbs from light, and this energy, which is used by cells, is supplemented in an inverse relationship with the energy provided by ATP; 2. there is a causal link between melanin, infection, and neurodegenerative diseases; 3. reduction in melanin levels alters the energy supply of cells in disease, which results in a lack of energy to sustain immune system defenses and remove the plaques seen in neurodegenerative diseases; and 4. the loss of neuromelanin in neurodegenerative diseases may be due to the same amyloid fibril bleaching process that takes place in the extracellular ß-amyloid and/or may be due to uptake of melanin by pathogens that use host melanin to fortify their own immune systems and/or for other expenditures. We do not think the effect or significance of melanin is limited to the diseases discussed. In fact, we theorize that melanin is the unifying factor underlying disease states. Future studies should further investigate this linkage and develop more sophisticated models of our understanding of these and other diseases. This could lead to new insights, a better understanding of disease, the immune system, how pathogens may be utilizing the host immune systems, not only to invade, but to possibly fortify their attacks, and new treatments for diseases.

## Data availability statement

The original contributions presented in the study are included in the article/supplementary material. Further inquiries can be directed to the corresponding authors.

## Author contributions

SB was responsible for the conception of the original theory, interpretation of the published research used to substantiate the work, drafting the work, and making critical revisions to the work. JB was responsible for the conception of the understanding of the mitochondrial underpinnings and other substantial contributions to the work, including interpretation of previous research used to substantiate the work, drafting a part of the work, and making critical revisions to the work. All authors contributed to the article and approved the submitted version.
